# Assessment of macular function, structure and predictive value of pattern electroretinogram parameters for postoperative visual acuity in patients with idiopathic epimacular membrane

**DOI:** 10.1007/s10633-016-9543-0

**Published:** 2016-04-28

**Authors:** Wojciech Lubiński, Wojciech Gosławski, Karol Krzystolik, Maciej Mularczyk, Leszek Kuprjanowicz, Michał Post

**Affiliations:** 1 Clinic of Ophthalmology, Pomeranian Medical University, Powstańców Wlkp. 72 Str., 70-111 Szczecin, Poland; 2 Pomeranian Medical University, Szczecin, Poland

**Keywords:** Epimacular membrane, VA, PERG, OCT

## Abstract

**Purpose:**

To evaluate macular function and structure before and after epimacular membrane surgery and to estimate the usefulness of pattern ERG test parameters in predicting the postoperative visual acuity.

**Methods:**

We evaluated 32 eyes of 32 patients (mean age 70.8 ± 6.7 years) before and 12 months after successful 25G pars plana vitrectomy with epimacular membrane removal and internal limiting membrane peeling. Distance best-corrected visual acuity (DBCVA—logMAR), foveal thickness (optical coherence tomography—OCT) and macular function [pattern electroretinogram—PERG (ISCEV standard): amplitudes (A) of P50- and N95-waves, implicit time (IT) of P50-wave] were assessed. To estimate the differences between the mean values of considered characteristics, the t test or Wilcoxon matched pair test was used. Correlation between preoperative data of PERG and preoperative and final DBCVA were investigated using Pearson correlation analysis. A receiver operating characteristic curve was constructed to obtain a cutoff value allowing prediction of visual prognosis. We tried to obtain the P50 and N95 amplitudes cutoff value in prediction of good visual outcome (DBCVA of 0.3 or less).

**Results:**

Twelve months after surgery, mean of DBCVA significantly increased in comparison with preoperative value (0.31 ± 0.12 vs. 0.6 ± 0.15; *p* < 0.001) and 23/32 eyes (72 %) achieved visual improvement of two and more Snellen lines. In OCT test, the significant reduction in foveal thickness mean (313.34 ± 47.01 vs. 509.03 ± 93.88 µm; *p* < 0.001) was obtained. In PERG test, the significant increase in the mean amplitudes of P50- (AP50) and N95 (AN95)-waves as well as significant decrease in the mean implicit time (IT) of P50-wave were achieved (AP50: 3.41 ± 1.48 vs. 2.38 ± 1.23 µV; *p* < 0.001; AN95: 5.46 ± 1.72 vs. 3.75 ± 1.48 µV; *p* < 0.001; IT P50: 55.00 ± 3.60 vs. 56.75 ± 5.78 ms; *p* < 0.001). Twelve months postoperatively, DBCVA was significantly correlated with preoperative IT P50 (*r* = 0.39; *p* = 0.027), AP50 (*r* = −0.68; *p* < 0.001) and AN95 (*r* = −0.73; *p* < 0.001).

**Conclusion:**

Removal of idiopathic epimacular membranes with internal limiting membrane peeling not only provided increase in visual acuity and reduction in foveal thickness but also caused improvement of innermost retinal layer function in macular region. Pattern ERG test might be a valuable tool in predicting the postoperative visual acuity.

## Introduction

Idiopathic epimacular membranes (EMMs) are hypocellular, largely collagen structures caused by glial proliferation through a defect in the internal limiting lamina usually in process of posterior vitreous detachment [1]. Their prevalence increases significantly with age, from <1 % in people under 50 years to 15 % or more in those over 70 years of age [2]. Treatment of EMMs includes pars plana vitrectomy (PPV) and membrane peeling (MP), with about 80 % of patients achieving visual acuity (VA) improvement of two or more lines and reduction in metamorphopsia [3].

Success of the EMMs treatment is usually assessed by visual acuity test and optic coherent tomography (OCT) imaging. Multifocal electroretinogram (mfERG) has been used to investigate the physiological changes in eyes with idiopathic EMM [4–7]. The results of these studies suggested that this test can objectively show macular dysfunction not limited only to the fovea but might also be a valuable tool in predicting the postoperative visual function. However, mfERG originates from preganglionic elements [8], so this test does not evaluate function of ganglion cells of the macular region. The bioelectrical function of these cells, which can be also abnormal in patients with EMMs, can be assessed by pattern PERG [9]. In the available literature, only one study described macular function using PERG and focal electroretinogram (fERG) before and after epimacular membrane surgery, but with no International Society for Clinical Electrophysiology of Vision (ISCEV) standards [10]. It was shown that decrease in visual acuity is related to dysfunction of preganglionic and ganglionic macular elements and that surgical removal of EMM may induce functional improvement of outer and inner retinal layers connected with increasing of visual acuity.

The goal of present study was to evaluate macular function and structure before and after epimacular membrane surgery and to estimate the usefulness of ISCEV standard pattern ERG parameters in predicting the postoperative visual acuity.

## Patients and methods

Thirty-two eyes of 32 selected, pseudophakic patients (mean age 70.8 ± 6.7 years; 10 men and 22 women) were included in this study. The clinical diagnosis of EMM was confirmed by patient’s complaints, indirect binocular ophthalmoscopy (Volk Optical +90 D non-contact lens) and OCT (an increase in macular thickness with optically hyperreflective epiretinal echoes from membrane). Patients presenting poor cooperation and with ocular and systemic diseases with known influence on the PERG results were excluded from these study.

Electrophysiological results from EMM eyes were compared with normative data obtained from 30 eyes of 15 healthy subjects. The controls were matched so there were no significant differences between age, sex and refractive errors between them and examined patients.

Before and 12 months after surgery the following examinations were performed: distance best-corrected visual acuity (DBCVA) using logMAR—ETDRS chart, foveal thickness (Zeiss Cirrus OCT, Humphrey Instruments model 3000, Carl Zeiss Inc., Dublin, California) and transient PERG according ISCEV standards [11].

Transient PERG was recorded with the RetiPort (Roland Consult Instr.) system. Protocol of the PERG test was implemented in the original software of the system. Patient’s pupils were not dilated, monocular stimulation was used, refraction correction was applied with respect to the eye–screen distance 0.5 m, and interruptions of the test were introduced when frequent blinking or fixation loose was observed (patient was monitored with a TV camera).

Parameters of the pattern stimulation were as follows: 21″ CRT monitor with a frame rate equal to 75 fps was used; black and white reversing checkerboard (30° Field of View) was presented to the patient, with a check size equal to 1°2′; luminance for white elements: 120 cd/m^2^, contrast: 97 %; temporal frequency was equal to 4.6 rps (2.3 Hz); and central fixation was used. Thread DTL electrode was used as active, and gold disk was placed at the ipsilateral outer canthus as reference, with ground (gold disk) electrode placed on the forehead (Fpz).

Parameters of the recording system were as follows: amplifiers sensitivity: 20 μV/div, filters: 1–100 Hz. Notch filters: off. Artifact reject threshold: 95 % (for the amplifiers range ±100 μV). Sweep time: 250 ms (time base: 25 ms/div). Average: 200 sweeps. Two consecutive waveforms were recorded, off-line averaged and then analyzed.

According to the standard, amplitude as well as time parameters of the obtained waveforms were analyzed; manual correction was applied to the automatic cursors placement. Values of all parameters were compared with the own laboratory normal values.

### Surgical procedure

Surgical procedure consisted of 25G three-port pars plana vitrectomy by two experienced surgeons (WL, LK) with complete removal of EMM by retinal forceps with peeling of internal limiting membrane (ILM) after Trypan Blue dye injection. After vitrectomy SF6 gas to posterior eye segment was introduced.

### Statistical analysis

To estimate the differences between the mean values of considered characteristics, the t test or Wilcoxon matched pair test was used (Statistica 10 software). Correlation between preoperative PERG data and preoperative and final DBCVA were investigated using Pearson correlation analysis. A receiver operating characteristic (ROC) curve was constructed to obtain a cutoff value allowing prediction of visual prognosis. We tried to obtain amplitude of P50 and amplitude of N95 cutoff value in prediction of good visual outcome (DBCVA of 0.3 or less). The level of statistical significance was set at *p* < 0.05.

All patients signed written consent form in regard to the PERG examination (not necessary in regular follow-up of patients after vitrectomy). The study was approved by the Pomeranian Medical University Research Ethics Committee.

## Results

The epimacular membrane was successfully removed from macular region in all treated eyes. There were no serious intraoperative or postoperative complications.

The clinical characteristics of EMM eyes before and 12 months after surgery are presented in Table 1.

**Table 1:** Clinical characteristics of EMM eyes before and 12 months after surgery

Patient	Age (years)	DBCVA (logMAR)		Foveal thickness (µm)		PERG					
						P50 amplitude (µV)		N95 amplitude (µV)		P50 implicit time (ms)	
		Baseline	12 months	Baseline	12 months	Baseline	12 months	Baseline	12 months	Baseline	12 months
1	66	0.52	0.08	438	284	3.64	4.22	5.68	6.43	51	54
2	52	0.48	0.12	508	280	4.99	6.21	6.04	6.95	53	54
3	71	0.24	0.14	393	270	4.01	6.67	5.89	7.39	51	52
4	65	0.52	0.22	448	270	3.23	5.53	5.68	7.91	59	58
5	63	0.22	0.12	354	272	4.47	5.67	5.65	7.32	50	51
6	70	0.62	0.24	640	311	3.97	4.34	5.65	5.85	54	53
7	68	0.54	0.26	483	283	3.83	5.79	5.34	8.81	56	53
8	74	0.64	0.32	458	318	3.32	3.98	5.21	6.15	58	60
9	73	0.52	0.18	424	246	4.86	4.82	5.06	6.24	50	52
10	76	0.72	0.38	575	350	3.78	4.44	4.83	5.83	57	55
11	68	0.42	0.24	320	243	2.17	2.76	4.52	5.85	55	56
12	61	0.48	0.12	393	279	2.32	3.31	4.47	8.85	56	51
13	69	0.78	0.34	653	298	2.22	2.86	4.26	5.24	48	50
14	66	0.68	0.32	616	321	2.28	3.46	4.14	5.22	57	55
15	74	0.64	0.26	559	343	2.13	4.74	4.12	8.03	54	54
16	74	0.48	0.34	425	308	2.19	2.96	4.12	5.62	53	52
17	74	0.52	0.32	524	350	2.17	4.01	4.04	5.92	62	58
18	74	0.62	0.36	679	344	2.11	3.04	3.91	4.12	54	57
19	80	0.52	0.26	485	378	1.75	2.86	2.98	5.61	60	55
20	68	0.56	0.22	580	288	1.02	3.17	2.93	6.07	54	52
21	67	0.58	0.34	447	388	1.07	2.98	2.92	4.32	46	51
22	84	0.72	0.42	594	354	1.18	2.08	2.58	4.41	67	59
23	75	0.78	0.54	668	392	1.28	1.85	2.44	3.51	59	60
24	82	0.64	0.48	589	386	1.46	1.98	2.16	3.94	62	58
25	81	0.68	0.36	587	368	1.15	2.04	1.96	3.41	68	59
26	63	0.52	0.32	580	351	1.48	1.78	1.94	4.49	66	58
27	75	0.84	0.36	488	266	1.34	2.72	2.02	4.34	64	60
28	63	0.74	0.32	512	382	1.22	1.77	1.98	4.28	66	60
29	74	0.68	0.52	456	272	1.08	1.24	2.04	2.69	55	50
30	73	0.76	0.46	487	226	1.43	1.65	1.87	2.63	50	47
31	72	0.86	0.54	524	313	1.02	1.85	1.67	2.29	63	59
32	70	0.64	0.28	402	293	1.86	2.47	2.04	4.94	58	57

Twelve months after surgery, statistically significant improvement was observed in logMAR DBCVA, foveal thickness and PERG parameters (*p* < 0.001).

### DBCVA

Twelve months after surgery, mean of logMAR DBCVA improved significantly compared with baseline (*p* < 0.001) and 23/32 eyes (72 %) achieved improvement of visual acuity of two and more Snellen lines. About 65 % (21/32) of eyes achieved final VA of 0.32 or less (0.5 or more Snellen).

### Foveal thickness

The mean preoperative foveal thickness was 509.03 ± 93.88 µm (range 320–679 µm). After surgery significant reduction in foveal thickness was obtained and was equal 313.34 ± 47.01 µm. In only one eye, foveal thickness 12 months after surgery was between normal limits (212 ± 20 µm).

### PERG

In PERG test performed after surgery, the mean implicit time of P50-wave significantly decreased in comparison with preoperative value (*p* < 0.001), (Table 2). Postoperatively, shortening of IT of P50-wave was detected in 20/32(62.5 %) of operated eyes (mean 4 ms) and in 13/20 (65 %) of eyes exceeding 5 % of initial values. Before surgery, prolonged IT of P50-wave was observed in nine eyes in comparison with range of normal values established in our laboratory (46.5–59.2 ms). After the surgery return to normal limits was seen in 7/9 of the eyes (77.7 %),

**Table 2:** Mean values and standard deviations in group of 32 eyes of patients before and 12 months after EMM surgery

Trait	EMM surgery	Mean	SD	*p* Value
logMAR DBCVA	Before	0.60	0.15	<0.001*
	After	0.31	0.12	
Foveal thickness	Before	509.03	93.88	<0.001*
	After	313.34	47.01	
IT P50	Before	56.75	5.78	<0.001*
	After	55.00	3.60	
A P50	Before	2.38	1.23	<0.001*
	After	3.41	1.48	
A N95	Before	3.75	1.48	<0.001*
	After	5.46	1.72	

The differences between means were statistically significant in all cases

* Differences statistically significant

Postoperatively, the mean amplitude of P50-wave significantly increased in comparison with baseline (*p* < 0.001; Table 2). After surgery, increase in amplitude of P50-wave was detected in 96.8 % (31/32) of eyes (mean 1.01 µV) and in 25/32 (78 %) of eyes it was exceeding 20 % of initial values. Before surgery, reduced amplitude of P50-wave was seen in 68.7 % (22/32) of eyes in comparison with range of normal values in our laboratory (3.2–11.3 µV). After surgery, return to normal values was achieved in 4/22 of the eyes (18.2 %).

**Fig. 1:**
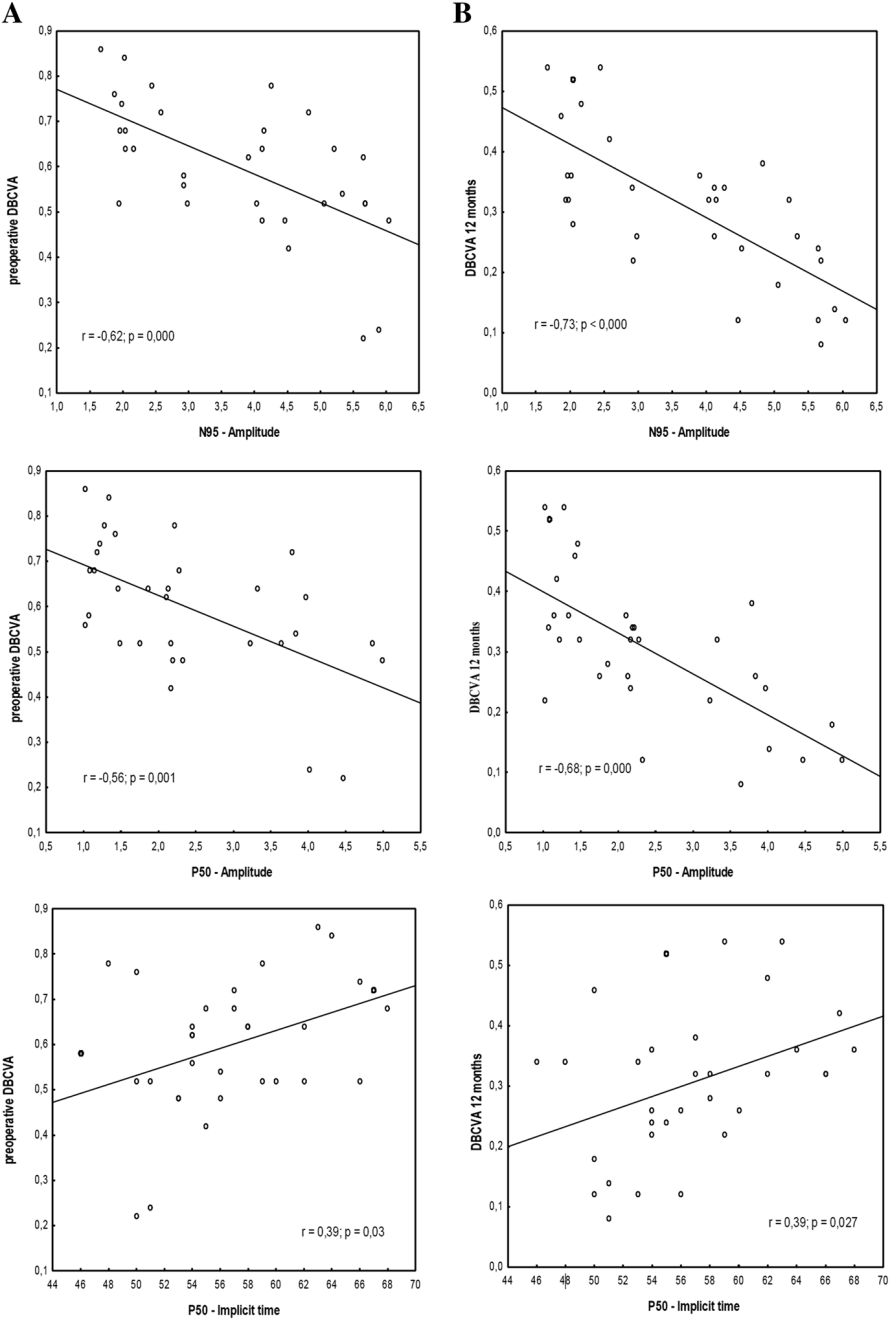
*Scatter plots* of preoperative DBCVA and preoperative PERG values in patients with EMM (**a**), and DBCVA 12 months after surgery and preoperative PERG values in patients with EMM (**b**). Significant correlations between preoperative and 12 months after surgery DBCVA versus preoperative PERG values were obtained

Twelve months after surgery, the mean amplitude of N95-wave also significantly increased in comparison with baseline value (*p* < 0.001; Table 2). After surgery increase in amplitude of N95-wave was observed in all cases (mean 1.64 µV) and in 25/32 of eyes (78 %) it exceeded by 20 % initial values. Before surgery, reduced amplitude of N95-wave was seen in 68.7 % (22/32) of eyes in comparison with normal values in our laboratory (4.8–15.7 µV). Postoperatively return to normal values was detected in 10/22 of the eyes (45.5 %). Correlations between preoperative and 12 months after surgery DBCVA versus preoperative PERG values are shown Fig. 1.

**Fig. 2:**
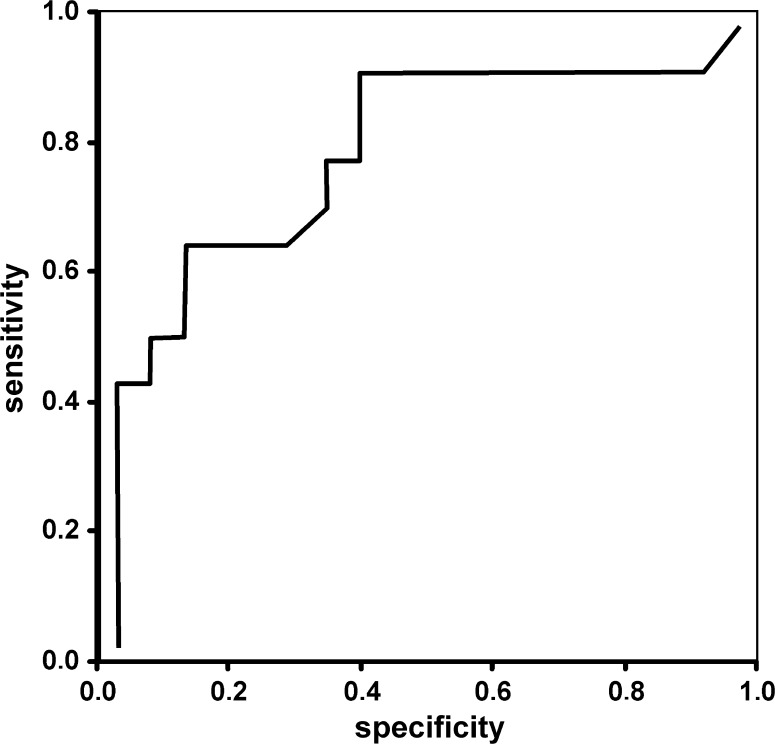
ROC curve for preoperative P 50 amplitude used as a prognostic factor for good postoperative DBCVA of 0.3 logMAR or less (0.5 or better Snellen). The cutoff point = 2.32 µV; specificity = 64.3 % sensitivity = 88.9 % and AUC = 0.81

**Fig. 3:**
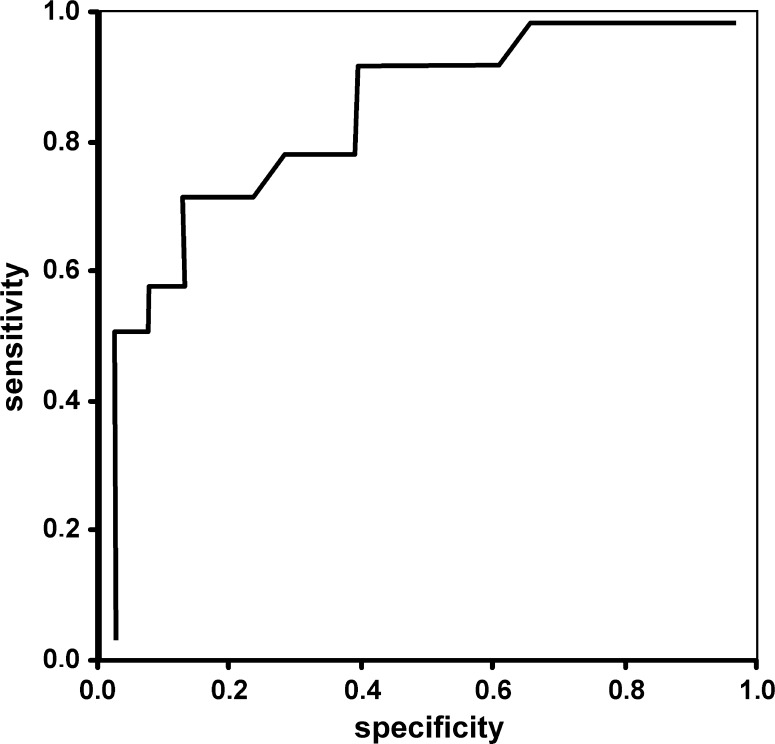
ROC curve for preoperative N 95 amplitude used as a prognostic factor for good postoperative DBCVA of 0.3 logMAR or less (0.5 or better Snellen). The cutoff point = 4.47 µV; specificity = 71.4 %; sensitivity = 88.9 % and AUC = 0.86

**Fig. 4:**
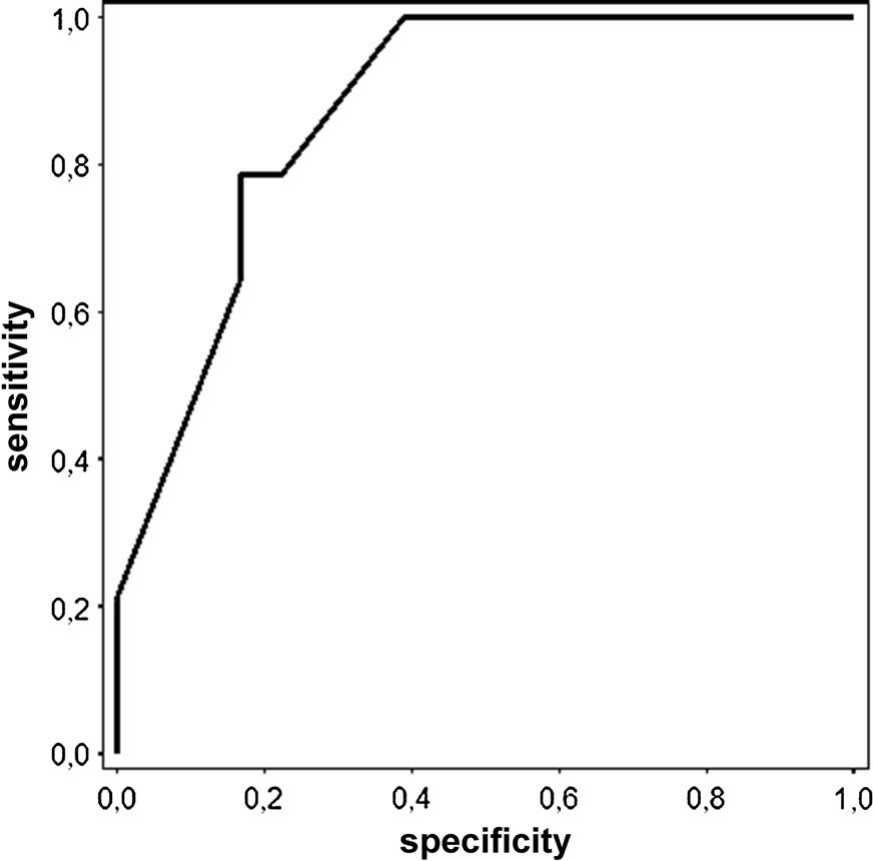
ROC curve for preoperative DBCVA (logMAR scale) used as a prognostic factor for good postoperative DBCVA of 0.3 logMAR or less (0.5 or better Snellen). The cutoff point = 0.56; specificity = 83.3 %; sensitivity = 78.6 % and AUC = 0.87

Figures 2, 3 and 4 show ROC curves for P50 and N95 waves and preoperative DBCVA used as a prognostic factor for good postoperative DBCVA of 0.3 or less (0.5 or better Snellen).

The areas under the ROC curve (AUC) for A P50 was 0.81. The cutoff value for good VA was 2.32 µV (sensitivity = 88.9 %, specificity = 64.3 %).

The area under the ROC curve (AUC) for AN95 was 0.86. The cutoff value for good VA was 4.47 µV (sensitivity = 88.9 %, specificity = 71.4 %).

The area under the ROC curve (AUC) for preoperative DBCVA was 0.87. The cutoff value for good VA was = 0.56 (sensitivity = 78.6 % specificity = 83.3 %). The AUC for the preoperative DBCVA was the highest, but the difference is relatively small and statistically insignificant.

**Fig. 5:**
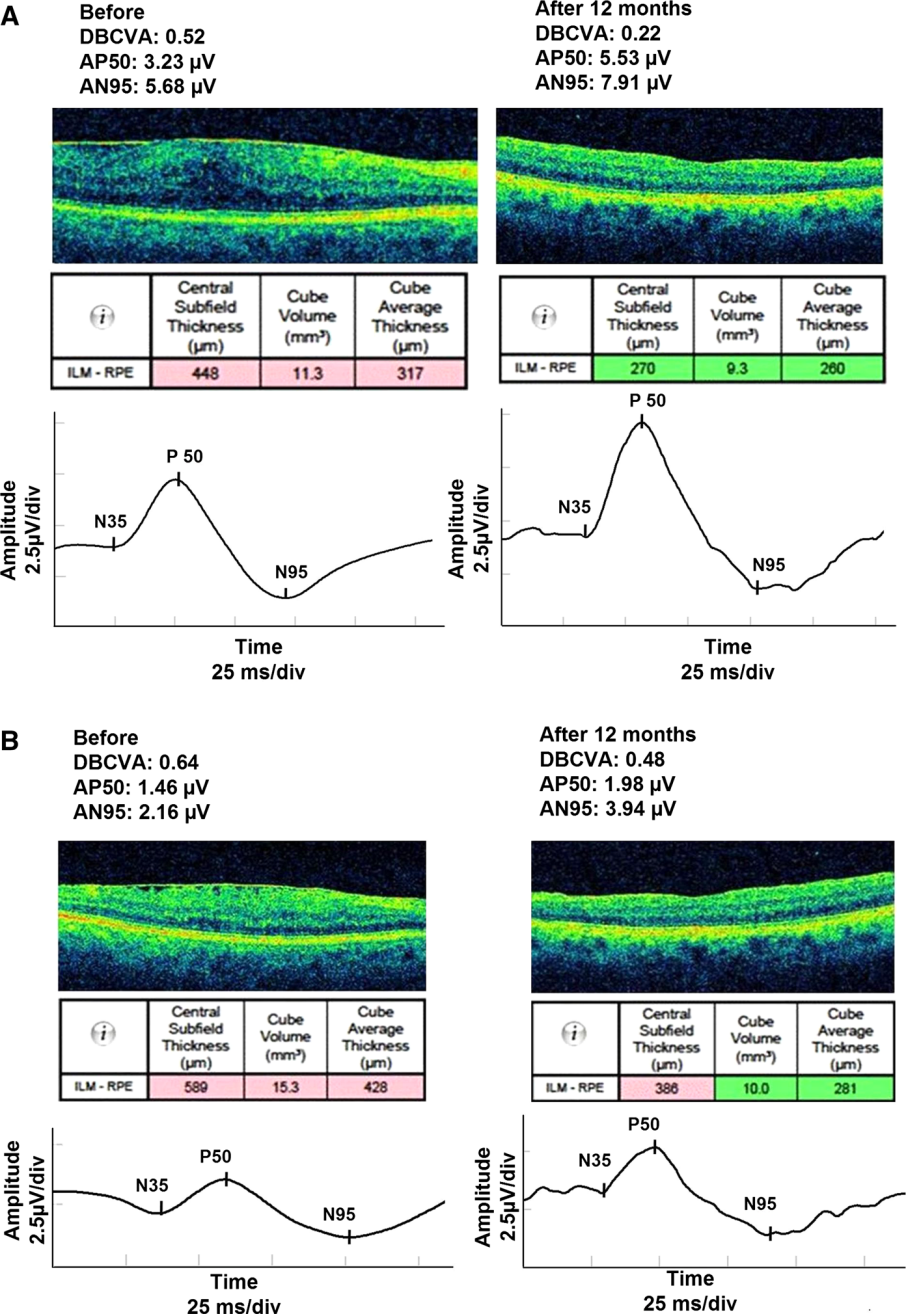
A 65-year-old woman with initial VA of logMAR 0.52. (Table 1—patient 4). One year after surgery increase in amplitudes of P50- (from 3.23 to 5.63 µV)and N95-waves (from 5.68 to 7.91 µV) waves was associated with foveal thickness reduction and improvement of DBCVA to logMAR 0.22. *Note* before surgery, the amplitudes of P50- and N95-waves were higher than cutoff values for these waves obtained from ROC curve. 5B 63-year-old woman with initial VA of logMAR 0.64 (Table 1 patient 24). One year after surgery, small increase in amplitudes of P50- (from 1.46 to 1.98 µV) and N95-waves (from 2.16 to 3.94 µV) waves was associated with foveal thickness reduction and small improvement of DBCVA to logMAR 0.48. *Note* before surgery, the amplitudes of P50- and N95-waves were below than cutoff values for these waves obtained from ROC curve

Representative PERG and OCT results of two patients with different visual prognosis after EMM surgery are shown in Fig. 5a, b. Preoperative VA for first and second presented patient were logMAR 0.52. (Snellen 0.3) and logMAR 0.64 (Snellen 0.23), accordingly. The postoperative VA logMAR 0.22 (Snellen 0.6) and logMAR 0.48 (Snellen 0.33), corresponding to 3 and 1 Snellen line gain, accordingly. Preoperatively amplitudes of P50- and N95-waves were above in the first patient but below in the second patient than cutoff values for these waves obtained from ROC curve indicating possible predictive role of electrophysiology testing.

## Discussion

In eyes with EMM, the several factors (e.g., incomplete posterior vitreous detachment) may induce cell proliferation in retinal inner limiting membrane. In EMM different cell types such as astrocytes, fibrocytes, macrophages, hyalocytes were found [12, 13] and some of them have characteristics of myofibroblasts [14]. The consequences of these events are macular distortion and/or macular edema and axoplasmic stasis. The manifestations of macular edema and or mechanical traction by EMM were reduction in DBCVA, retinal functional impairment [10] and increase in foveal thickness seen in OCT what is consistent with other study results [15].

Before surgery all eyes with EMM had reduced DBCVA, increase in foveal thickness and in majority of eyes (69 %) reduced amplitudes of P50- and N95-waves in comparison with normal values from the control group. The increase in IT of P50-wave) was obtained in 28 % of eyes. Visual acuity reduction correlated with the PERG preoperative parameters.

PERG derives from retinal ganglion cells and neighboring inner retinal structures [9, 16] not specified more precisely. The data from several investigators suggest that the N95 component of the human PERG is a contrast-related component generated in relation to the retinal ganglion cells. P50 originates partially from ganglion cells and also distally to the ganglion cells, from cells that are not yet ascertained [17]. Delayed implicit time of P50-wave suggests inner retinal dysfunction distally to the ganglion cells layer.

In our study, the detection of PERG abnormalities (reduced amplitudes of P50- and N95-waves) in EMM eyes indicates that dysfunction is present in ganglion cells layer and also in the inner retinal layers distally to the ganglion cells layer (delayed IT of P50-wave). The results are consistent with of Parisi et al. [10] study. The occurrence of functional impairment of outer retinal layer in patients with EMM was also documented by multifocal ERG or focal ERG in the other studies [4–7, 10, 18]. However, in some patients selective dysfunction of innermost macular layers was detected [10]. The cause of the PERG abnormalities may be macular edema as well as traction of ganglion cells layer by EMM, leading clinically to reduction in visual acuity.

Twelve months after surgery, significant increase in DBCVA, reduction in foveal thickness, increase in amplitudes of P50- and N95-waves and decrease in P50 implicit time were observed (Table 2). Seventy-two percent of eyes achieved visual acuity improvement of two and more Snellen lines. About 65 % of eyes achieved final DBCVA of 0.32 or less logMAR (0.5 or better Snellen). The obtained results are consistent with results from other studies that reported success rate of 70–80 % after surgery estimated by VA improvement [18–20].

The postoperative mean foveal thickness was reduced but was still higher than upper limit of normal value what is with agreement with other study results [21].

In PERG test, in the majority of operated eyes (78 %) the most prominent change was significant [22] increase in P50- and N95-waves amplitudes exceeding 20 % of initial values. It is worth noting that most often postoperative improvement of N95 amplitude was achieved (45.5 % of eyes). In patients with initially prolonged IT of P50-wave, shortening of IT was observed in 78 % of eyes, postoperatively. Postoperative DBCVA significantly correlated with the PERG preoperative parameters (Fig. 1).

The results of our study suggest that significant improvement of DBCVA, reduction in foveal thickness and improvement of PERG parameters were associated with postoperative partial restoration of macular structure and function (reduction in edema and mechanical traction of ganglion cells and their fibers by EMM). It seems reasonable to conclude that innermost retinal layers may have significant influence on macular function recovery after pars plana vitrectomy with EMM and ILM peeling. Our results are consistent with the results Parisi et al. [10].

ROC curves for amplitudes of P50- and N95-waves were used to establish cutoff values for good postoperative DBCVA of 0.3 or less (0.5 or better Snellen), (Figs. 2, 3).

The areas under curve (AUC) (commonly used predictor for postoperative acuity) were statistically significant when amplitudes of P50- and N95-waves were evaluated and indicate that these parameters may be useful to identify patients with favorable visual prognosis in clinical practice. Although, the AUC for preoperative DBCVA (Fig. 4) was higher than for PERG amplitudes, but the difference was not statistically significant and it may be affected by limited group size. To eliminate the bias, further studies with a larger number of eyes should be conducted.

Many prognostic factors for postoperative visual acuity after EMM surgery were analyzed in previous studies [21], but only preoperative VA was the variable consistently associated with postoperative VA. In these studies, IS/OS integrity in OCT was probably associated with postoperative VA. It was possible that severity of metamorphopsia, cone outer segment tips integrity and fundus autofluorescence also influenced postoperative VA. Central foveal thickness was a variable not consistently associated with postoperative VA. The studies with adequate methodological quality are necessary to confirm these findings. We think that PERG parameters should also be included in the overall prediction model as it has been shown that multiple regression models including both presurgical acuity and PERG amplitudes P50 (*R*^2^ = 0.65) or N95 (*R*^2^ = 0.67) explain better postsurgery acuity (in 65 and 67 %, respectively) than simple regression with: presurgery acuity *R*^2^ = 0.55; or presurgical PERG amplitudes P50 *R*^2^ = 0.46 or N95 *R*^2^ = 0.53. Although presurgical visual acuity remains slightly but statistically insignificant), stronger predictor of the postsurgical acuity compared to PERG amplitudes, PERG amplitudes have also significant predictive value and application of both parameters presents better predictive value as shown in example presented in Fig. 5a, b.

In conclusion, removal of idiopathic epimacular membranes with internal limiting membrane peeling not only provided visual acuity increase and reduction in foveal thickness but also was a cause of innermost retinal layer function improvement in macular region. Pattern ERG test might be a valuable tool in predicting the postoperative visual acuity.

Further study with larger group of patients should be performed to confirm our presented results.
